# Study on the Strength and Micro Characteristics of Grouted Specimens with Different Superfine Cement Contents

**DOI:** 10.3390/ma14216709

**Published:** 2021-11-08

**Authors:** Dongyue Zhang, Zhenqian Ma, Yihuai Zou, Hongfei Xie, Ruichong Guan

**Affiliations:** 1 School of Mining, Guizhou University, Guiyang 550025, China; 17885906234@163.com (D.Z.); yhzou@gzu.edu.cn (Y.Z.); zhoulang20161941@163.com (R.G.); 2 Guizhou Panjiang Coal and Electricity Group Technology Research Institute Co., Ltd., Guiyang 550081, China; zqwei1996@163.com

**Keywords:** superfine cement, uniaxial compression test, fractal dimension of debris, mercury intrusion porosimetry, scanning electron microscopy

## Abstract

To provide the most effective comprehensive performance grouting material ratio, in this experimental investigation, a total of eight grouted specimens with two water-cement ratios (0.45:1, 0.55:1) and four different superfine cement contents (0%, 30%, 70%, 100%) were evaluated. Based on a uniaxial compression test, the fractal dimension of the fragments, a mercury injection test, and scanning electron microscopy, the effects of the superfine cement content on the strength characteristics and microscopic characteristics of the grouted specimens were studied. The results showed that increasing the superfine cement content could enhance the compressive and tensile strength of the grouted specimens and reduce the fractal dimension of the fragments and the porosity of the grouted specimens. The superfine cement content increased from 0% to 70% when the water-cement ratio was 0.45:1. The compressive strength of the grouted specimens increased from 16.7 MPa to 26.3 MPa, and the fractal dimension decreased from 1.8645 to 1.2301. When the water-cement ratio was 0.55:1, the compressive strength of the grouted specimens increased from 10.5 MPa to 20.6 MPa, and the fractal dimension value decreased from 2.2955 to 1.4458. When the superfine cement content increased from 0% to 100%, the water-cement ratio was 0.45:1. The porosity of the grouted specimens was reduced from 28.41% to 21.62%. When the water-cement ratio was 0.55:1, the porosity of the grouted specimens was reduced from 33.33% to 29.46%.

## 1. Introduction

With the continuous development of roadway support theory, grouting support has been widely used in roadway to control surrounding rock. Previous engineering practice has shown that the use of Portland cement as a grouted material can enhance the bearing capacity of surrounding rock [1,2], which illustrates the fact that grouting has great significance in the stability control of roadway surrounding rock. In this connection, superfine cement is a high-performance grouting material that is composed of high-strength cement, expansion agent, slag, and other additives, and is relied upon because it has superior permeability, pourability, and higher strength and durability than ordinary cement [3,4]. Superfine cement has been widely used in the field of roadway surrounding rock grouting and remarkable results have been achieved.

In the research on superfine cement grouting, scholars around the world have used physical experiments, theoretical analysis, numerical simulation, and other methods to study the physical properties and microstructure of grouting with different compositions, and they have achieved fruitful results. For example, J.J. Chen [5] added superfine cement into Portland cement slurry, and the changes in bulk density, water film thickness, compressive strength, and water separation rate of the cement slurry were studied. J. Kaufmann [6] studied the effects of superfine cement and short fiber on the shrinkage, rheological, and mechanical properties of Portland cement slurry. Wenguang Jiang [7] experimentally analyzed the influence of nano-SiO_2_ on the viscosity, mechanical properties, and microstructure of superfine cement. Xiong Zhang [8] added superfine additives to cement paste, and the effect of superfine additives on the rheological properties of cement paste were studied. Zhang Lei [9] used nano silica sol to modify the mechanical properties of superfine cement slurry and performed orthogonal experiments to analyze the influence of different nano silica sol content on the strength of silicate slurry. Yang Qianrong [10] studied the effects of the water-cement ratio, bone:cement ratio, mineral admixtures, and chemical additives on the rheological properties of Portland cement slurry. Eyubhan Avci [11] analyzed the effects on the strength and permeability of various graded sand samples when using superfine cement and fine fly ash as grouting materials. The results showed that the addition of fine fly ash to superfine cement grouts accelerated the grouted specimens’ strength gain. Moreover, in Eyubhan Avci’s other experimental research [12], the effect of silica fumes on the grouting performance of superfine cement and the engineering properties of grouted sand were examined. Wenshuai Li [13] evaluated the effects of the particle sizes of cement and nano-SiO_2_ on shear strength parameters, microstructure characteristics, and rheological properties. The results showed that with the addition of superfine cement and nano-SiO_2_, the early-age shear strength parameters and microstructure characteristics of the specimens were significantly improved.

Furthermore, researchers have used methods including direct shear experiments, theoretical analysis, and numerical simulation to study the shear properties of rock mass [14], Portland cement grouting specimens [15,16], grouted fractured rock mass [17] and the interface between rock and tailings backfill [18], and have found that grouting could effectively change the shear properties of these specimens. Although many scholars have evaluated the comprehensive performance of Portland cement and superfine cement grouting specimens, there have been few studies on the influence of superfine cement content on the mechanical properties, microstructures, and pore size distributions of slurry casting specimens. Therefore, in light of the previous studies on the influence of grouting on rock shear properties, in this research, different proportions of superfine cement and ordinary (Portland cement) cement were mixed, and then the grouted specimens were poured to carry out a uniaxial compression test, Brazilian splitting test, mercury intrusion test, and scanning electron microscopy. The pore size distribution, micro crack structure characteristics, and mechanical parameters of grouted specimens with different cement ratios, were analyzed to determine the optimal grouting material ratio.

## 2. Indoor Experiments

### 2.1. Experimental Plan Design

In this experimental investigation, the brand of Portland cement was CONCH, with the model number P.C 32.5. This cement is generally used as the main material of roadway grouting in Guizhou Province, China. The brand of superfine cement was KAMABELLA and the model was CGM 800. This brand is widely used in many kinds of soft rock roadways in Guizhou because of its excellent permeability. It can effectively control broken surrounding rock when it is used as a grouting material. Some parameters of the two cements given by the merchant are shown in Table 1 below.

After determining the cement brand model, the superfine cement and Portland cement were mixed, and four different superfine cement and Portland cement ratios were set up. At the same time, based on on-site grouting experience, two water-cement ratios, 0.45:1 and 0.55:1, were used. There were four types of superfine cement content for the grouted specimens, 0%, 30%, 70%, and 100%. The Portland cement and the superfine cement were weighed separately and mixed into different experimental cement samples according to the ratios set in the plan, which were numbered as specimens #1 to #8. The experimental scheme is shown in Table 2.

According to different preset water-cement ratios, eight groups of experimental cement samples were injected with different qualities of water. The slurry was then fully stirred by a mixer and poured into the molds. Then, the abrasive tool was placed above the vibrating device to shake out the air bubbles in the slurry and reduce the artificial pores of the grouted specimens as much as possible. After the slurry had completely solidified, the mold was removed, and the grouted specimens were taken out. The two circular sections of the test piece were ground with a grinding machine. The cross-sectional flatness required an error of less than 0.02 m. Consistent with the requirements of Chinese national standard GB/T 50266-2013 [19] for rock mass test methods, the uniaxial compression test specimens were made into cylinders, and the uniaxial compression test specimens were cylindrical, with a diameter of 50 mm and a height of 100 mm. The Brazilian splitting test specimen was also cylindrical, with a diameter of 50 mm and a height of 25 mm. The grouted specimens were fabricated on the same day to avoid the error effect of cement aging on the experiment as much as possible.

The determination method for the uniaxial compressive strength of the grouted specimens was divided into the following steps: Firstly, the grouting slurry with different proportions of superfine cement was prepared using the mixing scheme described above and high-speed stirring with the mixer. Secondly, the grouting slurry was poured into molds with sizes of 50 mm × 100 mm and 50 mm × 25 mm × 100 mm and then solidified at 20 °C and 95% relative humidity for three days. Then the specimens were demolded. Next, the grouted specimens were subjected to uniform uniaxial loading experiments in a rock mechanics testing machine and loaded at 0.1 mm/min until the failure of the grouted specimens occurred; the mechanical parameters of the grouted specimens during the failure process were recorded. Each grouted specimen was subject to three groups of comparative tests.

**Figure 1:**
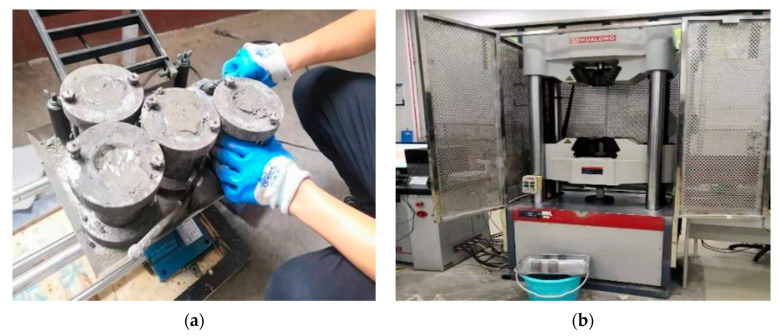
Pouring test piece site and rock mechanics testing machine. (**a**) On-site grouted specimens pouring. (**b**) Uniaxial experiment machine.

To ensure uniformity, each grouted specimen needed to be visually screened before the uniaxial experiment, and the grouted specimens with large surface bubbles needed to be discarded to meet the accuracy required by rock mechanics. The pouring of the on-site grouted specimens and the uniaxial experiment machine are shown in Figure 1.

### 2.2. Strength Characteristics of Grouted Specimens

**Figure 2:**
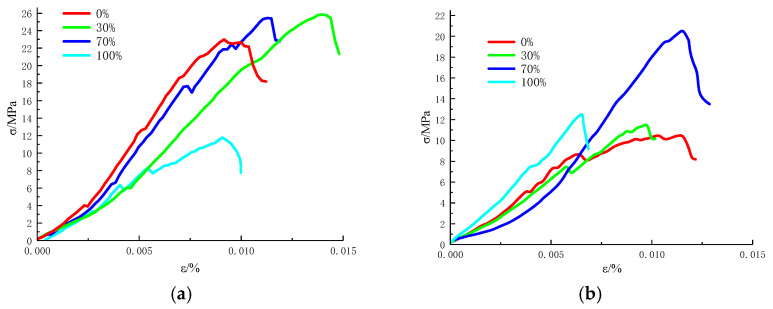
Uniaxial compression stress-strain curve of grouted specimens. (**a**) Water-cement ratio 0.45:1. (**b**) Water-cement ratio 0.55:1.

After the grouted specimens were prepared and perfected, they were tested with a uniaxial compressive strength split test using the WHY-5000 rock mechanics testing machine of Guizhou University. During the experiment, the loading rate was kept unchanged until the specimen was broken and, to reduce the error, three samples were set for each group of experiments. The obtained uniaxial compression stress-strain curve is shown in Figure 2, and the mechanical parameters of the grouted specimens are shown in Table 3.

The stress-strain curve is given by the experimental instrument. After the specimens were vertically fixed in the compression probe, the experiment started, the axial load increased and the specimens began to deform. To continuously fix the specimens, the compression probe moved downward, and the instrument determined the strain of the specimens by recording the downward movement distance of the compression probe during the uniaxial compression test. In the stress-strain curve, the unit of strain is %, which is the ratio of the deformation of the specimen to the original height of the specimens.

As can be seen from Figure 2, when the content of superfine cement of grouted specimens was 100% for the two water-cement ratios, the stress variables of the specimens were the smallest in the same group. When the material is damaged, low strain indicates that the brittleness of the material is high [20]. According to this hypothesis, the superfine cement was a material with high brittleness.

After mixing superfine cement with ordinary Portland cement, the brittleness of the grouted specimens was reduced and the elasticity of the specimens was improved. At the same time, the compressive strength of the grouted specimens was enhanced.

It can be seen from Table 3 that when the superfine cement content was less than 70%, increasing the superfine cement content could enhance the compressive strength and the tensile strength of the grouted specimens. This might have been due to the different sizes of ordinary cement particles and superfine cement particles. After the hydration reaction occurred when the two kinds of cement were mixed, the pores with different sizes in the grouted specimens were effectively filled, which could reduce the total porosity of the specimens, increasing the internal integrity of the grouted specimens and producing higher compressive and tensile strengths.

However, when the superfine cement content was further increased to 100%, the compressive strength and the tensile strength of the grouted specimens with two water-cement ratios decreased sharply, becoming the lowest of the four specimens in the same group. Consistent with the supposition that the superfine cement grouted specimens had high brittleness, it was shown that the superfine cement was a material with low strength and high brittleness. However, superfine cement usually has greater strength than ordinary cement. In the uniaxial experiment, the compressive strength of the superfine cement was lower than that of ordinary cement, which was probably because not enough water was being provided for the superfine cement in our experiment, resulting in an insufficient hydration reaction of the superfine cement. In addition, the uniaxial experiment result showed that the compressive and tensile strengths of the superfine cement specimen were higher when the water-cement ratio was 0.55:1, which provided support for the above conclusion. However, we only measured the uniaxial compressive strength of the ultra-fine cement and did not use other methods to evaluate the ultra-fine cement specimens, so the research results have limitations.

By comparing the mechanical parameters of grouted specimens for two different water-cement ratios, it could be concluded that when the superfine cement content was less than 70%, the compressive strength and the tensile strength of the grouted specimens with a water-cement ratio of 0.45:1 were higher than those for the grouted specimens with a water-cement ratio of 0.55:1. This showed that when the water-cement ratio was 0.55:1, the amount of water in the slurry was greater than the amount of water absorbed by the cement, and the remaining water evaporated while leaving pores in the grouted specimens, which reduced the strength of the grouted specimens. At the same time, this showed that there was an appropriate value of water content in the slurry ratio when the ordinary cement and superfine cement were mixed, which needed to be verified by many experiments.

## 3. Measure and Characteristic Analysis of Rock Debris

To further analyze the strength characteristics of grouted specimens with different superfine cement contents, we collected the cement debris from damaged cement specimens during uniaxial compression. After measuring the length, width, and height of all the cement debris and calculating the particle size with the formula, the fractal characteristics of the cement debris were analyzed. To reduce the error, each piece of debris was measured three times and the average value was taken. The results of the analysis were used to reveal the mechanical properties of cement specimens and the failure mechanism under a uniaxial load more fully and provide a theoretical basis for the research of grouting materials in roadway surrounding rock. In addition, they have significance for mine safety production and engineering research.

### 3.1. Analysis of Debris Size Characteristics

**Figure 3:**
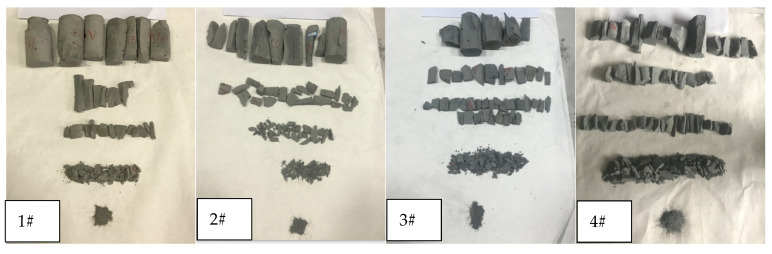

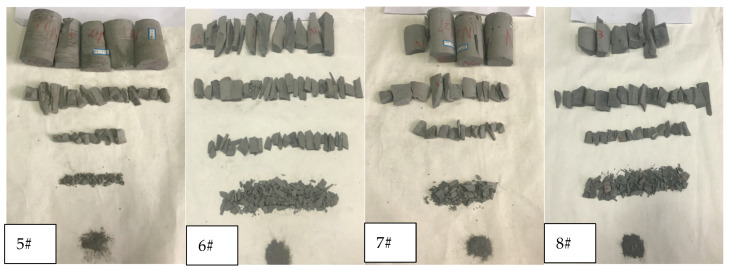
Fractal field of 1#–8# cement specimens.

**Figure 4:**
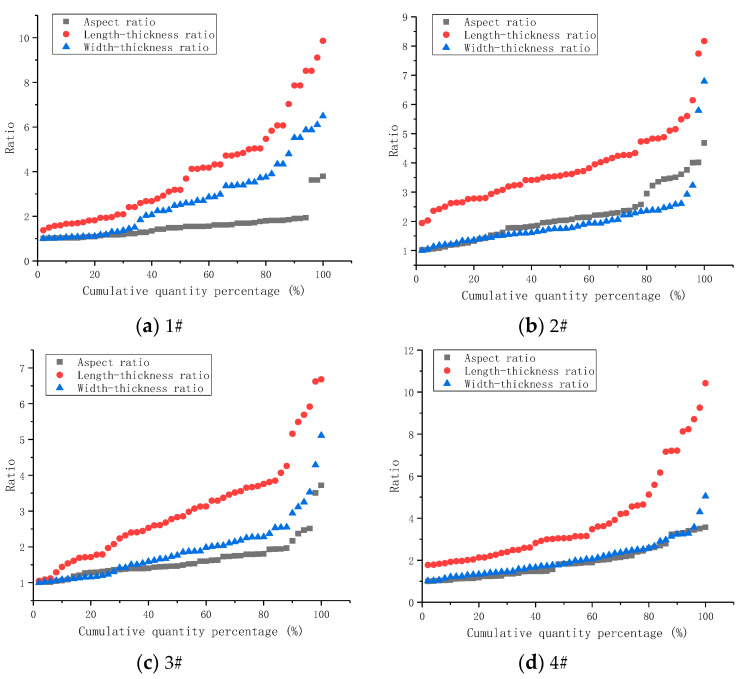
Detrital scale ratio distribution (water-cement ratio 0.45:1).

In this experiment, to obtain the length, width, height, and particle size for each piece of debris, the cement debris with particle sizes greater than 5.00 mm was counted, and the length, width, and thickness were measured with vernier calipers. At the same time, the aspect ratio, length-thickness ratio, and width-thickness ratio of the cement debris [21] were calculated, and for different superfine cement ratios; the ranges and average values of the aspect ratio, length-thickness ratio, and width-thickness ratio of the cement debris were as shown in Table 4. Figure 3 shows the size of debris after the grouted specimens broke. Figure 4 shows the comparison of the ratio distribution of the three directional scales (length, width, thickness) of cement debris.

When the largest sides of debris are placed horizontally downward, the maximum side length of the main view is defined as length, the second largest side length is defined as width, and the average height of the left view is defined as thickness. The aspect ratio is length/thickness, the width-thickness ratio is width/thickness, and the length-width ratio is length/width.

As shown in Figure 3, when the superfine cement content in the grouted specimens was low (Figure 3 (1#, 2#, 5#, 6#)), the failure mode of these grouted specimens was mainly axial splitting failure, and the failure degree of these specimens was insufficient. According to the results for the uniaxial compression specimens, the uniaxial compressive strength of these four specimens was relatively low. In the process of performing the uniaxial compression test, with increasing axial load on the grouted specimens, cracks began to develop in the specimens. However, when the content of superfine cement in the grouted specimens was low, the compressive strength of the specimen was also low and the cohesion was not strong enough, resulting in the cracks developing mainly along the stress direction with less circumferential diffusion. Under increasing load, the axial crack finally penetrated the specimens, the specimens were damaged and debris after damage mainly comprised long strips.

When the proportion of superfine cement in the grouted specimens was increased (As shown in the Figure 3 (3#, 4#, 7#, 8#)), according to the uniaxial compression test results, the uniaxial compressive strength of this grouted specimens was significantly increased. Therefore, when cracks developed inside the specimens, the stronger compressive strength and cohesion hindered the development of the cracks, so that the development direction of the cracks not only developed axially along the stress direction, but also spread around the interior of the specimens. Under increasing load, the cracks developed in multiple directions inside the specimens, which eventually lead to the failure of the specimen; the debris after the failure of the specimens was extensive, and the size distribution of the dust was more uniform.

**Figure 5:**
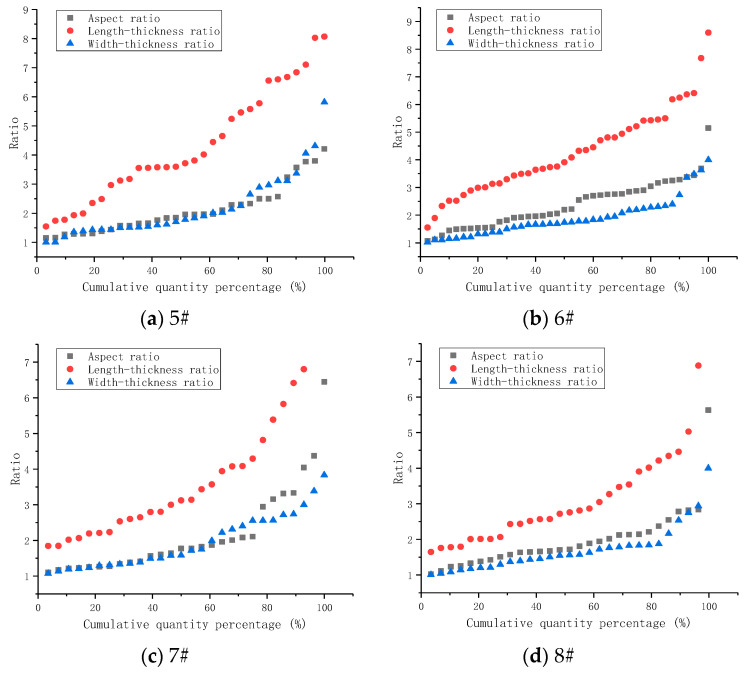
Detrital scale ratio distribution (water-cement ratio 0.55:1).

As shown in Figure 4 and Figure 5, and Table 4, the length-width ratio of the cement debris was nonlinear with the superfine cement content for the two kinds of water-cement ratios. When the content of superfine cement was increased to 30%, the length-width ratio of the cement debris increased. However, when the content of superfine cement was further increased to 70%, the ratio of length to width of the cement debris decreased. When the superfine cement content was 100%, the length-width ratio of the cement chips increased again.

When the superfine cement content of the grouted specimens increased from 0% to 70%, the length-thickness ratio and width-thickness ratio of the cement debris showed a negative correlation. When the water-cement ratio was 0.45:1, the length-thickness ratio of the grouted specimens decreased from 3.99 to 3.03. The width-thickness ratio decreased from 2.75 to 1.93. When the water-cement ratio was 0.55:1, the length-thickness ratio of the grouted specimens decreased from 4.31 to 2.62. The width-thickness ratio decreased from 2.18 to 1.56.

Since the loading mode of the strength test was uniaxial loading, the failure of grouted specimens was mainly manifested as longitudinal splitting failure, and there was less shear failure. Therefore, this section mainly describes the analysis of the length-thickness ratio of the grouted specimen debris. For the two water-cement ratios, when the content of superfine cement in the grouted specimens increased from 0% to 70%, the length-thickness ratio of the specimen’s debris decreased. This showed that increasing the content of superfine cement could make the debris size and the length-thickness ratio of the debris more uniform after the grouted specimens finally broke. When the length-thickness ratio of the grouted specimens was large, it indicated that the internal microcracks of the grouted specimens had continued to develop and penetrate during the stress load, and that the radiation range to the surrounding was small, which eventually led to the long strip structure of the cement debris.

Increasing the content of superfine cement in the grouting specimens could make the particle size of cement debris more uniform, indicating that the higher superfine cement content of the grouted specimens could improve the cohesion of the grouted specimens and enhance the comprehensive compressive capacity of the grouted specimens.

### 3.2. Debris Granularity-Number Fractal Dimension

**Figure 6:**
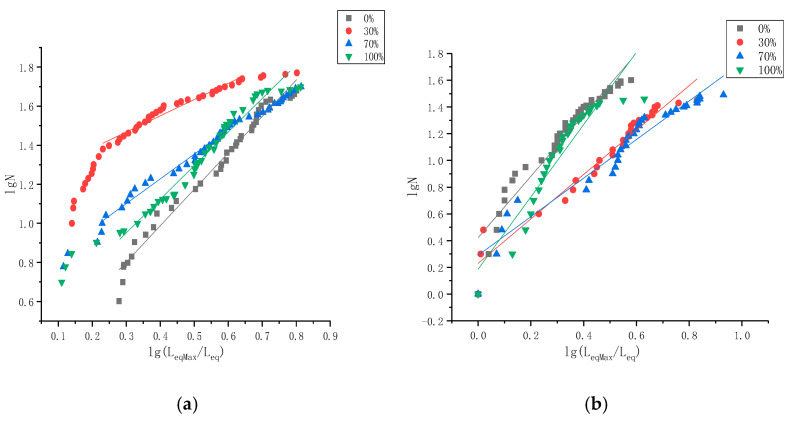
Granularity-number fractal dimension. (**a**) Water-cement ratio 0.45:1. (**b**) Water-cement ratio 0.55:1.

The fractal dimension of the debris with a particle size more than 5 mm was calculated. The measured length, width, and thickness values (l, w, h) of the cement debris were converted into the equivalent side length of an equivalent cube according to the following formula:

(1) $$N=N_{0}{\left(\frac{L_{\mathrm{eq}}}{L_{\mathrm{eqmax}}}\right)}^{-D}$$

where *L*_eq_ is the equivalent side length, which is (l × w × h)^1/3^; l, w, h are the length, width, and thickness of the cement debris, respectively; *N* is the number of debris pieces with an equivalent side length greater than the equivalent side length of the selected debris among all the debris; *N*_0_ is the number of debris pieces with the largest characteristic scale; and *D* is the fractal dimension. When lg*N* − lg (*L*_eqmax_/*L*_eq_) is used to draw a straight-line diagram, its slope is the fractal dimension [22,23], as seen in Figure 6 and Table 5.

As can be seen in Table 4, when the content of the superfine cement in the grouted specimens increased from 0% to 70%, both water-cement ratio grouted specimens debris fractal dimensions were decreased. The value of the fractal dimension of the grouted specimens with a water-cement ratio of 0.45:1 decreased from 1.8645 to 1.2301; the value of the fractal dimension of the grouted specimens with a water-cement ratio of 0.55:1 decreased from 2.2955 to 1.4458.

This was consistent with the law of performance in the strength determination of the grouted specimens, indicating that the data measured in the fractal dimension experiment was relatively accurate and had a degree of credibility. Consistent with fractal theory, when the cement specimens were broken, the fractal dimension of the debris was large, indicating that the size difference between each piece of cement debris was large and that the degree of damage to the specimens was incomplete. After the uniaxial compression test, the shape of the debris of the two same cement specimens should have been alike, and the value of the particle size for the quantity fractal dimension should have been similar. However, the fractal dimension of the grouting specimens with different superfine cement content varied greatly, indicating that the failure types of different specimens were completely different. The grouted specimens with a large fractal dimension showed that only part of the specimens had failed and were insufficient. Therefore, these cement specimens did not have the ability to deal with the uniaxial load, and the compressive strength was reduced. However, the grouting specimens with a large fractal dimension had the ability to fully deal with the uniaxial load in the process of uniaxial compression. The failure of the specimens was overall failure, and the compressive strength was improved.

In sum, increasing the superfine cement content of the grouted specimens could effectively slow down the crack growth rate of the grouted specimens under uniaxial compression, slowing down the damage of the grouted specimens and enhancing their compressive strength. At the same time, this reduced the breakage of the specimens after they were damaged.

As discussed in Section 2.1, it was shown that the hydration reaction of the superfine cement could provide the grouted specimens with a stronger cohesive force and cohesive force than ordinary cement. In theory, this indicated that the grouting slurry with high superfine cement content could better combine with the surrounding rock of the roadway after grouting, improve the comprehensive strength of surrounding rock, and better achieve the effect of stabilizing the surrounding rock. Since the uniaxial test could not restore the actual force state, the determination of whether this mixed slurry could enhance the stability of the roadway surrounding rock needed to be verified with engineering tests.

For the two water-cement ratios, the fractal dimensions of the grouted specimens with 100% superfine cement content were the largest in the same group. Consistent with the conclusion described in Section 2.1, it is suggested that the reason why the increase of the proportion of superfine cement in grouted specimens could improve the comprehensive strength of grouted specimens was that superfine cement could better combine different materials and provide stronger cohesion for the specimens to strengthen the specimens and improve the compressive strength.

## 4. Pore Analysis of Grouted Specimens

### 4.1. Porosity of Grouted Specimens

**Figure 7:**
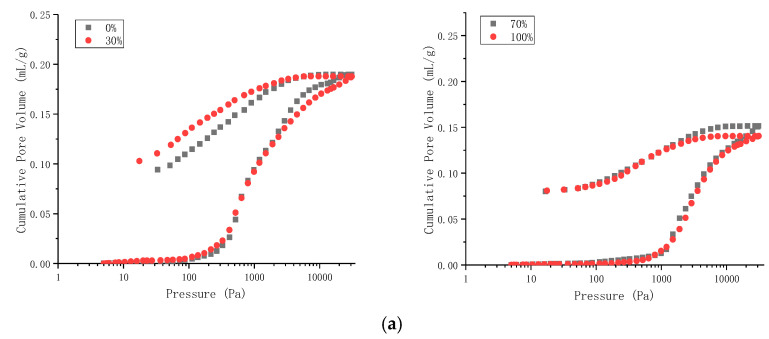

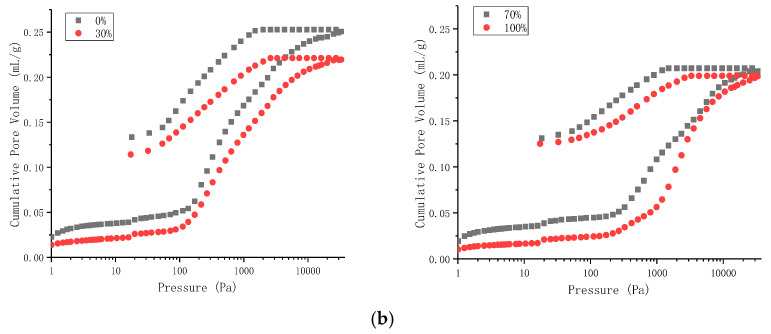
Advance and retreat mercury curve of grouted specimens. (**a**) Water-cement ratio 0.45:1. (**b**) Water-cement ratio 0.55:1.

The internal microstructure of the grouted specimens had direct effects on the macroscopic compressive and tensile strengths. Therefore, the mercury intrusion method was used to measure porosity of the grouting specimens. The principle of mercury intrusion porosimetry is to put mercury into the cracks of the sample by increasing the pressure of liquid mercury in a vacuum state. Mercury intrusion porosimetry was used to test the two water-cement ratio grouted specimens; the model of the mercury injection instrument was AutoPore IV 9500 (Micromeritics, Shanghai, China). The test results are shown in Figure 7.

It can be seen from Figure 7 that the mercury removal curves of the eight cement specimens under high pressure were relatively gentle, and there were more obvious retention loops. This result indicated that there were a large number of connected voids in the specimens. The data in Table 6 concern the porosity of the grouted specimens measured by mercury intrusion porosimetry.

From the data in Table 6, it can be seen that when the water-cement ratio was 0.45:1, the superfine cement content increased from 0% to 100%, while the porosity of the grouted specimens decreased from 28.41% to 21.62%. When the water-cement ratio was 0.55:1 and the superfine cement content was 30%, the porosity of the grouted specimens was the largest. When the superfine cement content was 100%, the porosity of the grouted specimens was the smallest. For the two water-cement ratios, the porosity of the grouted specimens with the superfine cement content of 100% was the smallest. This was because the particle size of the superfine cement was much smaller than that of ordinary Portland cement, and the smaller cement particles had a stronger invasion performance and could better fill the pores in the specimen. This resulted in the small porosity of grouting specimens with a high content of ultra-fine cement.

Comparing the porosity of the grouted specimens with two water-cement ratios, it was found that for the grouted specimens with the same superfine cement content, when the water-cement ratio was 0.45:1, the porosities of the grouted specimens were all less than the water-cement ratio of 0.55:1. This outcome was possible because the cement particles could not completely absorb the moisture, leading to some water remaining in the grouted specimens when the water-cement ratio was 0.55:1. After the water evaporated, pores were formed in the grouted specimens, which led to an increase in the porosity of the specimens.

According to the above results, it was determined that adding superfine cement to the grouting slurry could reduce the porosity of the slurry after solidification, and that this effect increased with the increase of the superfine cement content. To further analyze the reasons for this situation, the pore sizes of the grouted specimens were analyzed, as shown below.

### 4.2. Pore Size Analysis

**Figure 8:**
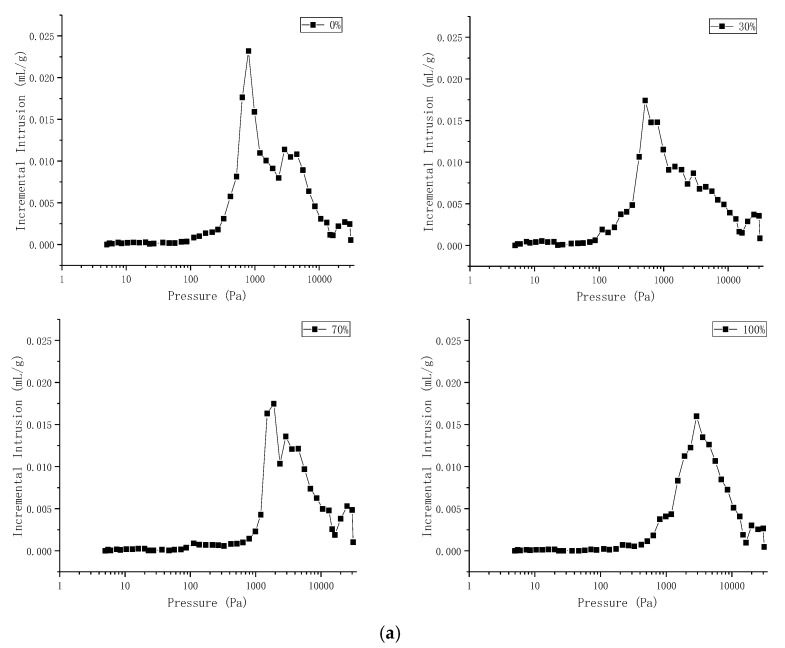

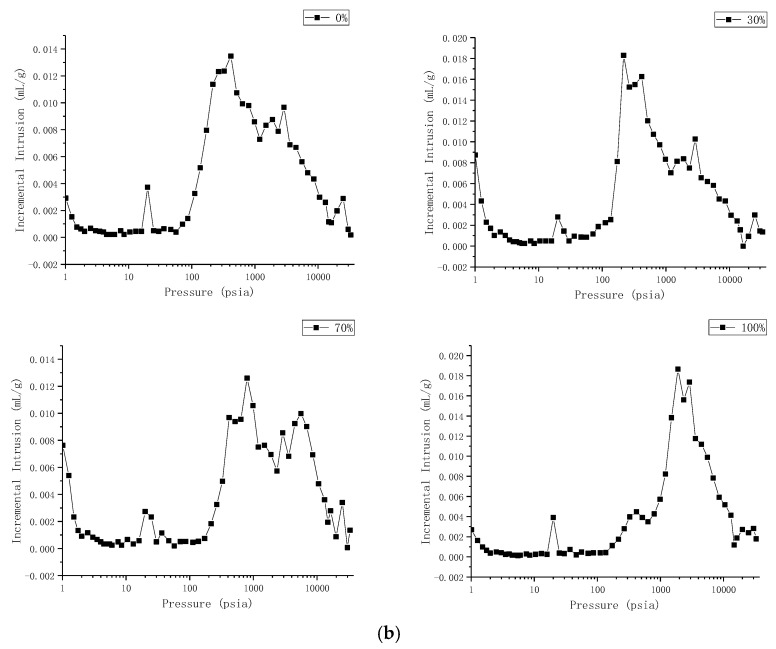
Grouted specimens pressure-incremental intrusion curve. (**a**) Water-cement ratio 0.45:1. (**b**) Water-cement ratio 0.55:1.

Table 7 shown the corresponding pressure at the peak of mercury intrusion during the mercury injection experiment of different grouted specimens. And the Figure 8 shown the pressure-incremental intrusion curve during the mercury injection experiment of different grouted specimens.

It can be seen from Figure 8 and Table 7 that for the two water-cement ratios, the grouted specimens with a low content of superfine cement, 1#, 2#, 5#, and 6#, had a large amount of mercury at the initial pressure (0–1000 Pa). The peak values of mercury intrusion were 798.15 Pa, 517.61 Pa, 416.53 Pa, and 216.50 Pa. This indicated that when the superfine cement content was low, there were many pores with larger diameters in the grouted specimens. After increasing the superfine cement content, the 3#, 4#, 7#, and 8# grouted specimens with a higher superfine cement content had a smoother mercury penetration curve at the initial pressure (0–1000 Pa). This indicated that there were fewer pores with larger inner diameters. After continuing to pressurize to 1000 Pa, the mercury ingress curve rate of the specimens increased rapidly, and the peak mercury intrusion amounts were 1897.37 Pa, 2894.88 Pa, 797.01 Pa, and 1897.40 Pa. This showed that the pore diameters in the specimens with a high content of superfine cement were smaller. This situation was probable because the superfine cement had better permeability than ordinary cement, and could penetrate into the pores with smaller diameters and fill the pores after the hydration reaction, thus reducing the pore diameters in the grouted specimens and finally reducing the total porosity of the specimens.

For the two water-cement ratios, the cement specimens with the lowest pressure, corresponding to the peak mercury intrusion value, were the specimens with the 30% superfine cement content, which indicated that the pore diameters in these specimens were the largest. The reason for this situation might have been that the proportion of superfine cement in the grouted specimens was not high enough, so the cement particles could not fit adequately. Hence, the pore diameter in the specimens could not be reduced, and the opposite effect was achieved.

Previous researchers have divided pores with pore diameters of >1000 nm, 1000–100 nm, 100–10 nm, and <10 nm into macropores, mesopores, small pores, and micropores. The pore volume ratios of the grouted specimens according to the above categorization are shown in Table 8.

It can be seen from the data in Table 8 that with the increase of superfine cement content, the internal pores of the grouted specimens for two water-cement ratios were transformed from macropores and mesopores to small pores and micropores. When the water-cement ratio was 0.45:1, the mesopore volume ratio decreased from 53.4% to 18.6% with the increase of the superfine cement content from 0% to 100%. The small pore volume ratio increased from 35.2% to 74.1%. When the water-cement ratio was 0.55:1, the mesopore volume ratio decreased from 47.3%% to 26.4% with the increase of the superfine cement content from 0% to 100%. The small pore volume ratio increased from 28.2% to 55.6%%. The pore size of the grouted specimens for the two water-cement ratios ranged mainly from middle-size holes to small holes, which indicated that the permeability of the superfine cement was optimal between 100 nm and 1000 nm.

## 5. Microstructure Characteristics

**Figure 9:**
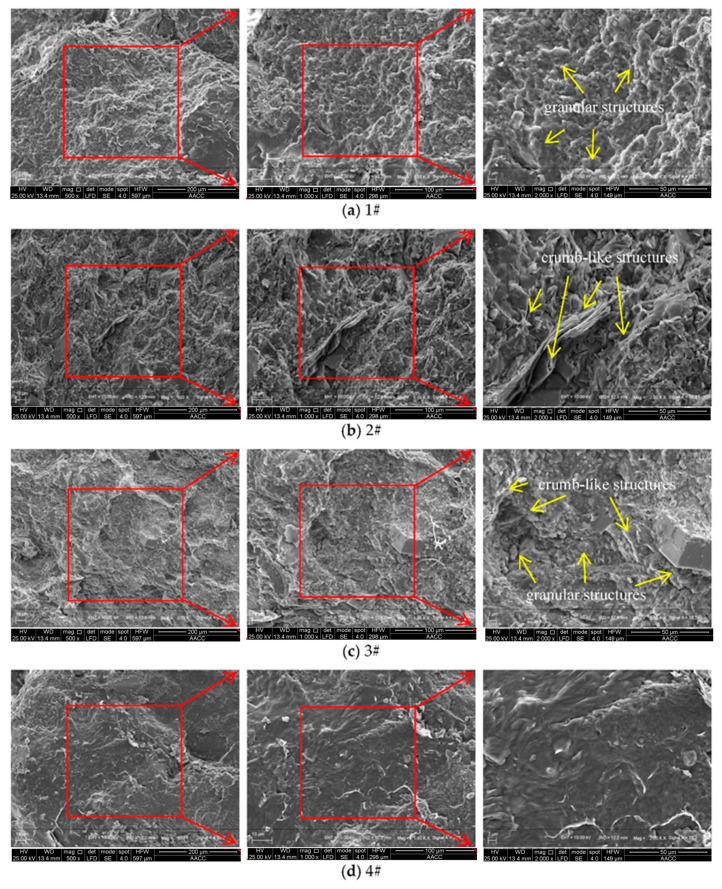
Scanning electron microscope microstructure of different specimens.

When the water-cement ratio was 0.55:1, the porosity of the grouted specimens was high, reflecting a more complex pore structure. When studying the influence of the proportion of superfine cement on the microstructure of the grouted specimens, these pore structures informed evaluation of the microstructure of the grouted specimens. The grouted specimens with a water-cement ratio of 0.45:1 were selected for scanning electron microscopy and the microstructure was analyzed. Figure 9 shows the scanning electron microscope microstructure of different grouted specimens at 500 times, 1000 times and 2000 times magnification.

It can be seen from Figure 9a that the grouted specimens with only ordinary cement had many granular structures on the surface, due to the large volume of ordinary cement particles. It can be seen from Figure 9b that after increasing the superfine cement content to 30%, the granular structure on the surface of the grouted specimens was significantly reduced. After the ordinary cement particles and superfine cement particles meshed with each other, many sharp crumb-like structures appeared. After further increasing the superfine cement content to 70%, it can be seen from Figure 9c that when the magnification was 500 times, the surface of the grouted specimens became smoother than that of the 1# specimens and 2# specimens. After further increasing the magnification, it was found that many particle structures and crumb-like structures coexisted. However, the size and length of the particle structure and the chip-like structure on the surface of the 3# test piece were far inferior to those of the 1# and 2# test pieces. The surface of the grouted specimens using only superfine cement shown in Figure 9d was very smooth, which was caused by the small particle size of the superfine cement.

## 6. Conclusion and Future Development

### 6.1. Conclusions

To provide a better grouting ratio, save material costs, and to improve the overall strength of grouting slurry, two groups of experimental specimens were designed with different mixes of ordinary cement and superfine cement. A total of eight grouted specimens with two water-cement ratios (0.45:1 and 0.55:1) and four different superfine cement contents (0%, 30%, 70%, and 100%) were produced. A uniaxial compression test, fractal dimension of the fragments, mercury injection test, and scanning electron microscope were applied to these grouted specimens, and the effects of the superfine cement content on the strength characteristics and microscopic characteristics of the grouted specimens were studied. The findings of this research were as follows.

(1) Superfine cement is a material with high brittleness. Mixing superfine cement with ordinary cement can reduce the brittleness and improve the elasticity of the grouted specimens. Additionally, superfine cement usually has greater strength than ordinary cement. However, in this research, the uniaxial compressive strength of superfine cement was lower than that of ordinary cement, which was probably because there was not enough water provided for the superfine cement in our experiment, resulting in an insufficient hydration reaction of the ultra-fine cement, and low strength was shown. In addition, the uniaxial experiment result showed that the compressive and tensile strengths of the superfine cement specimen were higher when the water-cement ratio was 0.55:1, which supported the above conclusion. However, we only measured the uniaxial compressive strength of the ultra-fine cement, and did not use other methods to evaluate the ultra-fine cement specimens, so the research results have limitations.

(2) After minimizing the influence of other factors on the experiment, we successively carried out mechanical experiments and fractal experiments on grouted specimens. We found that increasing the proportion of superfine cement in the grouted specimens could make the debris of the grouted specimens more uniform and narrow the ratio of length to thickness of the debris. This was conducive to the combination of different materials with superfine cement, which could provide stronger cohesion for the specimens and finally achieve the effect of strengthening.

(3) The pore diameter of the specimens with a higher content of superfine cement was smaller. This might have been because the superfine cement had better permeability than ordinary cement, and the superfine cement could penetrate the pores with smaller diameters and fill the pores after the hydration reaction, thus reducing the pore diameter in the grouted specimens and finally reducing the total porosity of the specimens. The pore size of the grouted specimens for the two water-cement ratios ranged mainly from middle-size holes to small holes, which indicated that the permeability of the superfine cement was optimal between 100 nm and 1000 nm.

(4) According to the above analysis, in this experiment, the grouting slurry with a water-cement ratio of 0.45:1 and a superfine cement content of 30–70% had better overall performance. However, because only two kinds of water-cement ratio and four kinds of superfine cement content slurry ratio experiments were designed in this research, better slurry ratio parameters still need to be found from a broader range of experiments.

### 6.2. Future Development

The results of this research demonstrated that the grouting slurry with a water-cement ratio of 0.45:1 and superfine cement content of 30–70% had better comprehensive performance. Since only two water-cement ratios and four kinds of superfine cement content were used in this experiment, the precision and reliability of the research requires to be improved. Therefore, we will use more precise tests in future research. For example, we intend to set the percentage difference of superfine cement content of each group of grouted specimens to 10%, and will study the comprehensive performance difference of different grouted specimens when the superfine cement content increases from 0% to 100%. Moreover, research into the water-cement ratio can be further refined. Better grouting ratio parameters need to be considered, using a wide range of tests, to further support the applicability of our research.

## Figures and Tables

**Table 1:** Some parameters of the two cements.

Cements	Compressive Strength (MPa)		Tensile Strength (MPa)	
	3 Days	28 Days	3 Days	28 Days
CGM800	23.7	37.1	2.8	3.1
P.C32.5	13.6	21.8	2.1	2.5

**Table 2:** Experimental program.

Specimens Number	Water-Cement Ratio	Percentage of Superfine Cement
1#	0.45:1	0%
2#		30%
3#		70%
4#		100%
5#	0.55:1	0%
6#		30%
7#		70%
8#		100%

**Table 3:** Mechanical parameters of grouted specimens.

Specimens’ Number	Water-Cement Ratio	Superfine Cement Content (%)	Compressive Strength (MPa)			Average Compressive Strength (MPa)	Tensile Strength (MPa)			Average Tensile Strength (MPa)
			1	2	3		1	2	3	
1#	0.45:1	0	17.1	16.9	16.1	16.7	1.97	2.36	2.42	2.25
2#		30	26.4	25.8	26.6	25.7	2.66	2.83	3.24	2.91
3#		70	23.8	27.5	27.6	26.3	4.01	3.83	3.68	3.84
4#		100	10.3	13.5	11.6	11.8	2.17	1.94	2.22	2.11
5#	0.55:1	0	11.6	11.3	8.6	10.5	1.88	2.01	1.96	1.95
6#		30	10.9	11.7	12.5	11.7	2.11	2.07	2.12	2.10
7#		70	18.4	21.3	22.1	20.6	2.35	2.69	2.91	2.65
8#		100	11.6	13.4	12.5	12.5	2.47	2.66	2.73	2.62

**Table 4:** Scale ratio of cement debris.

Specimens’ Number	Water-Cement Ratio	Superfine Cement Content (%)	Range of aspect Ratio/Average Value	Range of length-Thickness Ratio/Average Value	Range of width-Thickness Ratio/Average Value
1	0.45:1	0	1.01~3.80/1.57	1.38~9.86/3.99	1.00~6.49/2.75
2		30	1.02~4.68/2.22	1.94~8.17/3.83	1.00~6.79/2.00
3		70	1.01~3.72/1.64	1.00~6.68/3.03	1.02~5.11/1.93
4		100	1.00~3.57/1.92	1.78~10.42/3.90	1.01~5.04/2.07
5	0.55:1	0	1.15~4.21/2.10	1.55~8.07/4.31	1.01~5.82/2.18
6		30	1.07~5.15/2.38	1.55~8.60/4.28	1.02~4.00/1.91
7		70	1.01~3.35/1.48	1.27~5.15/2.62	1.01~2.84/1.56
8		100	1.05~4.10/1.89	1.28~6.50/2.83	1.03~3.87/1.72

**Table 5:** Granularity-number fractal dimension curve fitting.

Specimens’ Number	Water-Cement Ratio	Superfine Cement Content (%)	Curve Fitting	R^2^	Fractal Dimension
1#	0.45:1	0	y = 1.8645x + 0.2422	0.9774	1.8645
2#		30	y = 1.4275x + 0.5972	0.9420	1.4275
3#		70	y = 1.2301x + 0.2422	0.9850	1.2301
4#		100	y = 1.5540x + 0.2422	0.9678	1.5540
5#	0.55:1	30	y = 2.2955x + 0.4225	0.9256	2.2955
6#		70	y = 1.6619x + 0.1711	0.9527	1.6619
7#		0	y = 1.4458x + 0.2800	0.9302	1.4458
8#		100	y = 2.6038x + 0.1867	0.9036	2.6038

**Table 6:** Porosity of specimens.

Specimens’ Number	Water-Cement Ratio	Superfine Cement Content (%)	Porosity
1#	0.45:1	0%	28.41%
2#		30%	27.58%
3#		70%	22.72%
4#		100%	21.62%
5#	0.55:1	0%	36.81%
6#		30%	33.33%
7#		70%	31.44%
8#		100%	29.46%

**Table 7:** Peak mercury intrusion-corresponding pressure.

Specimens’ Number	Water-Cement Ratio	Superfine Cement Content (%)	Peak Mercury Intrusion (mL/g)	Corresponding Pressure (pa)
1#	0.45:1	0%	0.0232	798.15
2#		30%	0.0174	517.61
3#		70%	0.0175	1897.37
4#		100%	0.0160	2894.88
5#	0.55:1	0%	0.0135	416.53
6#		30%	0.0183	216.50
7#		70%	0.0126	797.01
8#		100%	0.0187	1897.40

**Table 8:** Pore volume ratio of slurry with different proportions.

Specimens’ Number	Water-Cement Ratio	Superfine Cement Content (%)	Pore Volume Ratio (%)			
			Macropores	Mesopores	Small Pores	Micropores
1	0.45:1	0%	3.2	53.4	35.2	5.8
2		30%	5.5	51.7	40.9	4.1
3		70%	3.0	19.1	68.0	9.9
4		100%	1.2	18.6	74.1	6.1
5	0.55:1	0%	21.4	47.3	28.2	3.1
6		30%	24.8	48.4	24.1	2.7
7		70%	22.7	37.7	37.4	2.2
8		100%	13.1	26.4	55.6	4.9

## Data Availability

This study did not report any data.

## References

[1] Lu Y., Wang L., Li Z., Sun H. (2017). Experimental Study on the Shear Behavior of Regular Sandstone Joints Filled with Cement Grout. Rock Mech. Rock Eng..

[2] Zhang W., Li S., Wei J., Zhang Q., Liu R., Zhang X., Yin H. (2018). Grouting rock fractures with cement and sodium silicate grout. Carbonates Evaporites.

[3] Li S., Sha F., Liu R., Zhang Q., Li Z. (2017). Investigation on fundamental properties of microfine cement and cement-slag grouts. Constr. Build. Mater..

[4] Stephanie Perret D.P., Gerard B. (2000). Rheological Behavior and Setting Time of Microfine Cement-Based Grouts. ACI Mater. J..

[5] Chen J., Kwan A. (2012). Superfine cement for improving packing density, rheology and strength of cement paste. Cem. Concr. Compos..

[6] Kaufmann J., Winnefeld F., Hesselbarth D. (2004). Effect of the addition of ultrafine cement and short fiber reinforcement on shrinkage, rheological and mechanical properties of Portland cement pastes. Cem. Concr. Compos..

[7] Jiang W., Li X., Lv Y., Jiang D., Liu Z., He C. (2020). Mechanical and hydration properties of low clinker cement containing high volume superfine blast furnace slag and nano silica. Constr. Build. Mater..

[8] Zhang X., Han J. (2000). The effect of ultra-fine admixture on the rheological property of cement paste. Cem. Concr. Res..

[9] Zhang L., Fan Z., Xi Kai Zhang S., Li Y. (2020). Study on properties of nano silica sol superfine cement based slurry. Min. Res. Dev..

[10] Yang Q., Zhao Z., Zhang Q., Li C. (2019). Influence of some factors on rheological properties of cement mortar. J. Build. Mater..

[11] (2020). Strength and Permeability Characteristics of Superfine Cement and Fine Fly Ash Mixture Grouted Sand. ACI Mater. J..

[12] Avci E. (2019). Silica Fume Effect on Engineering Properties of Superfine Cement–Grouted Sands. J. Mater. Civ. Eng..

[13] Li W., Shaikh F., Wang L., Lu Y., Wang B., Jiang C., Su Y. (2019). Experimental study on shear property and rheological characteristic of superfine cement grouts with nano-SiO2 addition. Constr. Build. Mater..

[14] Bahaaddini M., Sharrock G., Hebblewhite B. (2013). Numerical direct shear tests to model the shear behaviour of rock joints. Comput. Geotech..

[15] Tian H.M., Chen W.Z., Yang D.S., Yang J.P. (2015). Experimental and Numerical Analysis of the Shear Behaviour of Cemented Concrete–Rock Joints. Rock Mech. Rock Eng..

[16] Chen J., Hagan P., Saydam S. (2018). Shear behaviour of a cement grout tested in the direct shear test. Constr. Build. Mater..

[17] Salimian M., Baghbanan A., Hashemolhosseini H., Dehghanipoodeh M., Norouzi S. (2017). Effect of grouting on shear behavior of rock joint. Int. J. Rock Mech. Min. Sci..

[18] Fang K., Fall M. (2018). Effects of curing temperature on shear behaviour of cemented paste backfill-rock interface. Int. J. Rock Mech. Min. Sci..

[19] GB/T 50266-2013 (2013). Standard for Test Methods of Engineering Rock Mass.

[20] Zhou H., Meng F., Zhang C., Xu R., Lu J. (2014). Quantitative evaluation method of rock brittleness based on stress-strain curve. J. Rock Mech. Eng..

[21] He M., Yang G., Miao J., Jia X., Jiang T. (2009). Classification and research method of rock burst debris. J. Rock Mech. Eng..

[22] Shan X.N., Li Z. (2003). Fractal theory and Fractal Research on rock debrisation. J. Hebei Inst. Technol..

[23] Xie H., Peng R., Ju Y. (2004). Analysis of energy dissipation during rock deformation and failure. J. Rock Mech. Eng..

